# Grammatical Gender Disambiguates Syntactically Similar Nouns

**DOI:** 10.3390/e24040520

**Published:** 2022-04-07

**Authors:** Phillip G. Rogers, Stefan Th. Gries

**Affiliations:** 1 Department of Linguistics, University of California, Santa Barbara, CA 93106, USA; stgries@linguistics.ucsb.edu; 2 Department of English, Justus-Liebig University Giessen, 35390 Gießen, Germany

**Keywords:** syntax, grammatical gender, information theory, corpus linguistics, lexicon, usage-based

## Abstract

Recent research into grammatical gender from the perspective of information theory has shown how seemingly arbitrary gender systems can ease processing demands by guiding lexical prediction. When the gender of a noun is revealed in a preceding element, the list of possible candidates is reduced to the nouns assigned to that gender. This strategy can be particularly effective if it eliminates words that are likely to compete for activation against the intended word. We propose syntax as the crucial context within which words must be disambiguated, hypothesizing that syntactically similar words should be less likely to share a gender cross-linguistically. We draw on recent work on syntactic information in the lexicon to define the syntactic distribution of a word as a probability vector of its participation in various dependency relations, and we extract such relations for 32 languages from the Universal Dependencies Treebanks. Correlational and mixed-effects regression analyses reveal that syntactically similar nouns are less likely to share a gender, the opposite pattern that is found for semantically and orthographically similar words. We interpret this finding as a design feature of language, and this study adds to a growing body of research attesting to the ways in which functional pressures on learning, memory, production, and perception shape the lexicon in different ways.

## 1. Introduction

Grammatical gender has often been derided as an apparently arbitrary and unnecessary feature of language, perhaps most famously by Mark Twain in ‘The Awful German Language’ (1880): ‘In German, a young lady has no sex, while a turnip has. Think what overwrought reverence that shows for the turnip, and what callous disrespect for the girl…’ In languages with grammatical gender, nouns belong to two or more classes based on the agreement patterns they trigger in associated words. However, languages vary widely in their rules for assigning nouns to different genders [1] and these rules are often broken by conspicuous exceptions such as the ones highlighted by Twain.

Perhaps because of this reputation, linguists have long sought to understand what advantages grammatical gender might offer to language users. After all, how could such systems arise and persist in so many of the world’s languages if they served no purpose? For one, gender has been credited for linking temporally separated elements in discourse in languages with more flexible word orders such as Latin [2]. In a similar way, gender is thought to aid reference tracking in discourse by linking gendered anaphoric pronouns to the correct antecedent [3,4]. However, these explanations do not apply to all languages or even all cases of ambiguity [2].

Alternatively, accounts rooted in information theory continue to offer promising ideas concerning the functional advantages of gender. There are a number of psycholinguistic studies that suggest gendered articles can guide lexical prediction [5,6,7,8,9,10]. Some of these studies speak to the finer cognitive mechanisms underlying the boost to prediction such as the roles of facilitation and inhibition, but the general logic is straightforward: if the gender of a noun is revealed in a preceding element, the list of candidates that might fill that noun slot is reduced significantly. A recent corpus study of German provides empirical support for this theory, showing that gender marking on German articles serves to reduce the entropy (uncertainty) of upcoming nouns [2,11]. Adjectives may serve the same purpose in English, a language without gender [12]. These findings are consistent with information-theoretic predictions and research showing that speakers modulate speech in various ways to reduce excessive peaks and troughs in information density [13,14,15].

If reducing the possible set of candidate words is a general strategy for guiding lexical prediction, then a more direct strategy would be to target those candidates that are the most likely alternatives to the intended word. Put another way, the most efficient way to lower the uncertainty of an upcoming noun is to eliminate its strongest competitors. So, what kinds of nouns compete most strongly in lexical prediction?

One proposal suggests that it is semantically similar words that compete most strongly in this way. On the one hand, semantically similar words have been shown to cluster within genders across languages [1]. This is even true of inanimate nouns that fall outside of the semantically transparent semantic core of animate nouns [16]. On the other hand, exceptions abound, and these exceptions have been cited as evidence for the discriminatory role of gender. In a lengthy discussion of the complex relationship between semantics and gender assignment, Dye et al. [2] argue that the German gender system combines semantic clustering and semantic dispersal. If semantically similar nouns are largely clustered within genders, the assignment of some high frequency nouns to different genders would provide the most efficient reduction in entropy when gender tips its hand. The authors cite German words for drinks as an example. The words for beer (*Bier*) and water (*Wasser*) are neuter, while most other words for drinks in German are masculine (e.g., *Wein* ‘wine’, *Kaffee* ‘coffee’, *Tee* ‘tea’, etc.). Once the gender of a drink is revealed, listeners can safely eliminate either the two most predictable candidates or all the rest. Compare this scenario to one in which two low frequency drinks are the gender assignment exceptions; unless one of those two low frequency drinks are intended—which would be unlikely based on their low frequency—the reduction in entropy that comes with knowing the gender is minimal, only eliminating two candidates that were already improbable. In this way, semantic clustering of low frequency words and semantic dispersal of certain high frequency words can benefit discrimination. The authors found evidence for this kind of pattern across the German lexicon: High-frequency nouns tend to be distributed across genders in German, while low-frequency nouns tend to be clustered within the same gender.

Alternatively, one could argue it is phonologically similar words that compete most strongly in lexical prediction because they are potentially confusable, particularly from the perspective of noisy channel models [17]. However, it does not seem to be the case that gender discriminates such words. It is well known that gender is often marked phonologically on nouns [1]. The phonological rules for gender assignment vary widely from language to language, but within a given language, the nouns that share a particular diagnostic phonological pattern are overwhelmingly assigned to the same gender. To cite a familiar example, nouns in Spanish ending in *-o* are almost always masculine, while those ending in *-a* are almost always feminine. Therefore, it does not appear that grammatical gender disambiguates phonologically similar nouns.

In this paper, we argue that these previous accounts are actually missing a fundamental piece of the puzzle. It may not be very useful to ask whether gender helps to disambiguate semantically or phonologically similar words if one does not also control for *syntax*. We propose syntax as the locus of disambiguation because it represents the crucial context within which words must be discriminated. Nouns that tend to occur in the same syntactic contexts will compete for activation more than nouns that tend to occur in different syntactic contexts. Thus, if a primary function of grammatical gender is to guide lexical prediction, we hypothesize that nouns occurring in similar syntactic contexts should be less likely to share a grammatical gender. In this way, some of the strongest competitors of a target noun would be eliminated at the first indication of the word’s gender. Like some of the studies reviewed above, such a pattern would be probabilistic in nature, operating statistically across the lexicon, and yet it would constitute further evidence for functionally motivated structure underlying seemingly arbitrary grammatical gender systems.

To test this hypothesis, we must first define what is meant by the syntactic contexts of a word. This question may strike some linguists as odd given the traditional distinction in the field between lexicon and grammar. The former contains lexical items and their features that must be memorized, while the latter provides a finite set of rules allowing for theoretically infinite combinations of those lexical items. While most modern linguistic theories acknowledge that lexical items must be associated with *some* information about how they can (or cannot) be used in syntactic structures, there remains a reluctance in dominant frameworks to allow a richer integration between words and their syntactic structures. In modern generative theories, syntactic information in the lexicon is categorical (constraint-based), limited to rules concerning the syntactic frames in which a word can participate as a head or modifier [18,19,20,21,22,23,24]. These theories aspire to model language competence rather than performance [25], and as such see probabilistic aspects of language use as language-external and irrelevant to linguistic theory [26].

In contrast, we approach this from a usage-based perspective on language, which allows for a much richer representation of syntax in the lexicon. These theories posit that all aspects of language are connected in a cognitive network [27,28,29]. The strength of associative links between components of the network—such as words and syntactic structures—are based on one’s complex experience with them and related words and structures [27,30,31]. Importantly, this entails associations that are probabilistic in nature. From this perspective, words are situated in a rich, multi-dimensional space based on their features (e.g., phonological forms) and distributions (e.g., syntactic contexts).

There is growing evidence in usage-based research that distributional characteristics of words can impact language comprehension, production, and acquisition [32,33,34,35,36,37,38]. Of particular interest here are recent studies that provide a formal definition for the syntactic distribution of a word and demonstrate its predictive power in psycholinguistic datasets [39,40,41,42]. In these studies, the syntactic distribution of a word is defined as a probability distribution of the syntactic dependencies in which that word participates, where the dependencies refer to the asymmetric relations between ‘head’ and ‘dependent’ defined within Dependency Grammar formalisms [43,44,45,46]. This definition places words in a multidimensional syntactic vector space reminiscent of the distributional semantic spaces of computational linguistics, and it allows for fine-grained syntactic comparisons among words. The entropy of these syntactic distributions has been shown to correlate with production latencies and response times in lexical decision tasks [40,42], and syntactically similar words show priming effects [41].

Syntactic distributions defined in this way have also been tied to other grammatical phenomena. While previous studies have demonstrated a trade-off between syntactic and morphological complexity using word order (in-)flexibility to represent the contribution of syntax [47,48,49], a recent approach uses a new measure of syntactic complexity based on dependency relations: the aggregate uncertainty (entropy) of mapping from lexical items to syntactic function in a language, referred to as functional indeterminacy [50]. Across 44 languages, greater functional indeterminacy among nouns correlates with the presence of case marking and, for those languages with case systems, increased number of cases. This finding constitutes an empirical connection between a probabilistic representation of syntactic distributions and a well-known grammatical phenomenon.

The studies on syntactic distributions challenge us to reimagine syntax as a feature of words, on par with other word features such as semantic and phonological information. More concretely, they provide us with methods for precisely quantifying the syntactic distributions of words. In this paper, we use these insights to test our prediction that—across a large sample of languages—nouns will be assigned to genders such that gender supports the disambiguation of syntactically similar words. Put simply, syntactically similar words should be less likely to share gender than syntactically dissimilar words. Based on the literature reviewed above, we expect the opposite pattern for semantically and phonologically similar words.

## 2. Materials and Methods

The primary source of data for this study is the Universal Dependencies Treebanks (UDT) [51]. This project offers the cross-linguistically consistent part of speech tagging and dependency annotation for data from over 100 languages. Corpus size and the availability of additional features such as lemmas vary from language to language, and many languages are represented by multiple corpora. We extracted wordform, lemma, part of speech, gender, and syntactic information for every token of every corpus in UDT. For this study, languages without grammatical gender were excluded, as were corpora without consistent lemma information.

**Figure 1:**
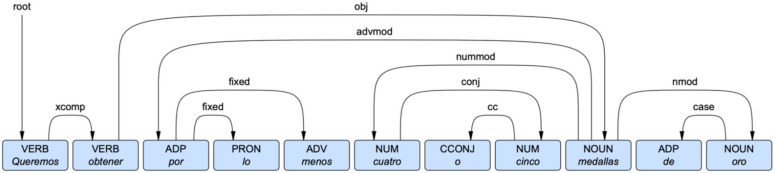
An example of the Universal Dependencies Treebanks dependency framework from Spanish. Syntactic dependencies are represented by arrows pointing from heads to their dependents, and each dependency is labeled for the type of relation. The translation of the sentence is ‘We want to get at least four or five gold medals’.

The syntactic information of a word consists of every syntactic dependency that the word participates in, either as a head or dependent. The UDT dependency framework is illustrated in Figure 1. In this example, the Spanish word *oro* (‘gold’) participates in two syntactic dependencies. First, it is the head of a case relation with *de* (‘of/from’). Second, it is the dependent of a nominal modification relation with *medallas* (‘medals’). These two relations highlight an important characteristic of the UDT framework: the primacy of content words. Practically speaking, this means UDT dependencies link content words directly rather than indirectly through function words. In contrast, many dependency grammars would view *oro* as a dependent of *de*, which in turn would be viewed as a dependent of *medallas*. For our purposes, we are interested in the overall syntactic distributions of words, so the particular framework by which those dependencies are annotated matters less than the consistency by which that framework is applied across sentences and languages.

Since grammatical gender is predominantly a feature of the lexeme rather than its specific wordforms, we aggregate the UDT data by lemma and part of speech. Upon aggregation, syntactic information takes the form of a syntactic vector. Each position in the vector represents a specific syntactic role and relation, such as the head of a determiner relation. The value at that position represents how many times a particular lemma was attested in that relation and role. As such, the entire vector constitutes a frequency distribution of the syntactic dependency types in which a lemma has participated.

Frequency distributions are known to be biased by sample size. Following Lester [42], we correct these distributions using the James–Stein shrinkage estimator [52]. This bias correction method performs well on data for which the number of types is known, and—given the size of our corpora—we assume that the dependency types represented by our corpus data are exhaustive. The bias correction also transforms the syntactic vector from a frequency distribution into a probability distribution. To ensure that the syntactic vectors included in the study are reliable, we exclude lemmas occurring less than ten times in our data.

**Figure 2:**
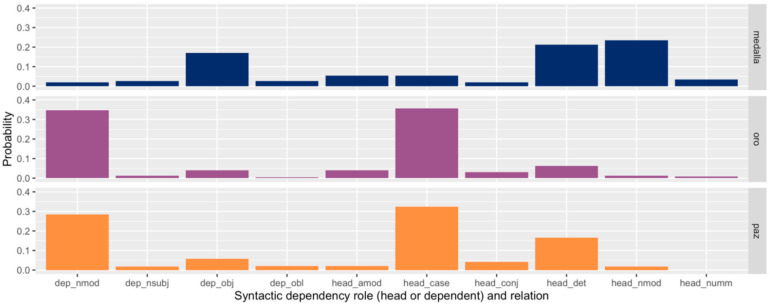
Partial probability vectors for the participation of three Spanish lemmas in different syntactic roles and relations. The height of each bar indicates how often that lemma participates in that dependency type relative to other syntactic dependency types. The probabilities shown are corrected for sample bias with the James–Stein shrinkage estimator. These three distributions illustrate how *oro* is much more similar syntactically to *paz* than to *medalla*, despite being more similar semantically to the latter.

We illustrate the syntactic distributions of lemmas with three examples from Spanish. Figure 2 shows partial probability vectors for three Spanish lemmas that are well attested in our data: *medalla* (*n* = 72), *oro* (98), and *paz* (132). The ten dependency types included in the illustration are only a subset of those found in Spanish, but they include types in which nouns often participate (they account for 87% of *medalla* dependencies, 93% of *oro*, and 96% of *paz*). The height of each bar represents the (bias-corrected) rate at which that lemma participates in that dependency type relative to other dependency types.

It is readily apparent from Figure 2 that *oro* and *paz* are much more similar to each other syntactically than either is to *medalla*. Both *oro* and *paz* participate frequently as the dependent in a nominal modifier dependency and as the head of a case marking dependency, while *medalla* does not. On the other hand, *medalla* is far more likely to occur as an object of a verb and as the head of a nominal modifier dependency. All four of these dependency types are illustrated with *oro* and *medalla(s)* in Figure 1. In fact, *oro* and *medalla* co-occur frequently in the corpus in the phrase *medalla(s) de oro* (‘gold medal(s)’), contributing to the patterns we observe in their syntactic distributions. These words—*oro* and *medalla*—are semantically similar yet syntactically distinct. In contrast, *oro* and *paz* are semantically unrelated yet syntactically similar.

To test our secondary hypotheses concerning the relationships of both semantics and orthography to gender sameness, we need semantic and phonological features for the lemmas in our study. The UDT corpora are too small to produce reliable semantic vectors, so fastText semantic vectors [53] are matched to words in the UDT. As the vectors from fastText correspond to wordforms, we compute weighted averages (by frequency) for lemmas to make them compatible with our data. Similarly, phonological transcriptions are not available for all the languages in our study, so we utilize orthography as a proxy for phonology. This is justifiable based on previous work: Dautrich et al. [54] examined the relationship between phonology and orthography and found high correlation between the number of phonemes and characters in a word in Dutch (*r* = 0.87), English (*r* = 0.83), German (*r* = 0.89), and French (*r* = 0.79).

### 2.1. Distance Measures

To assess the contributions of orthography, semantics, and especially syntax to gender sameness, we pair each noun lemma with every other noun in the language. This step offers two key advantages. First, we are not interested in predicting the gender of a given noun—for example, masculine or feminine. Rather, we are interested in predicting whether a given pair of nouns belong to the *same* gender, so each pair of nouns in the transformed data is coded for whether they share a gender. Second, pairing nouns allows us to reduce lengthy semantic and syntactic vectors and complex orthographic strings to the distance between two vectors/strings.

We use Levenshtein distance to represent the orthographic distance between two lemmas. Levenshtein distance is defined as the minimum number of single-character insertions, deletions, and/or substitutions needed to change one character string into another.

Cosine similarity is the standard metric for measuring similarity between two semantic vectors. This metric is popular because it captures the angle between the vectors in multidimensional space, ignoring the magnitude of those vectors. Subtracting the cosine similarity from 1 turns it into a distance metric. Given vectors *A* and *B*, where *A_i_* and *B_i_* are the components of these vectors, the formula for cosine similarity is:

(1) $$\frac{\sum_{i=1}^{n}A_{i}B_{i}}{\sqrt{\sum_{i=1}^{n}A_{i}^{2}}\sqrt{\sum_{i=1}^{n}B_{i}^{2}}}\;.$$

Finally, for syntactic distances, we follow Lester et al. [41] in using the entropy-based Jensen–Shannon Divergence (JSD) between syntactic vectors. JSD is a bounded, symmetric distance metric based on the Kullback–Leibler Divergence (KLD). KLD is an unbounded, directional (asymmetric) measure of the information loss of approximating one probability distribution by another, and JSD makes this measure bidirectional by averaging the distance to the midpoint of the two distributions. The relevant equations for JSD are as follows for probability distributions *P* and *Q* defined on the probability space *X*:

(2) $$\mathrm{JSD}\left(P∥Q\right)=\frac{1}{2}\mathrm{KLD}\left(P∥M\right)+\frac{1}{2}\mathrm{KLD}\left(Q∥M\right);$$

(3) $$\mathrm{KLD}\left(P∥Q\right)=\underset{x\in X}{\sum }P\left(x\right)\log\left(\frac{P\left(x\right)}{Q\left(x\right)}\right);$$

(4) $$M=\frac{1}{2}\left(P+Q\right).$$

### 2.2. Correlational Analysis

To assess the relationship between syntactic distance and gender sameness in pairs of lemmas, we first take a permutation approach. One straightforward way to perform such an analysis would be to permute the syntactic distances for a language in the paired lemma data and then calculate the correlation of this permuted variable with gender sameness. Performing this permutation many times would produce a null distribution of correlation values against which we could compare the real correlation.

**Figure 3:**
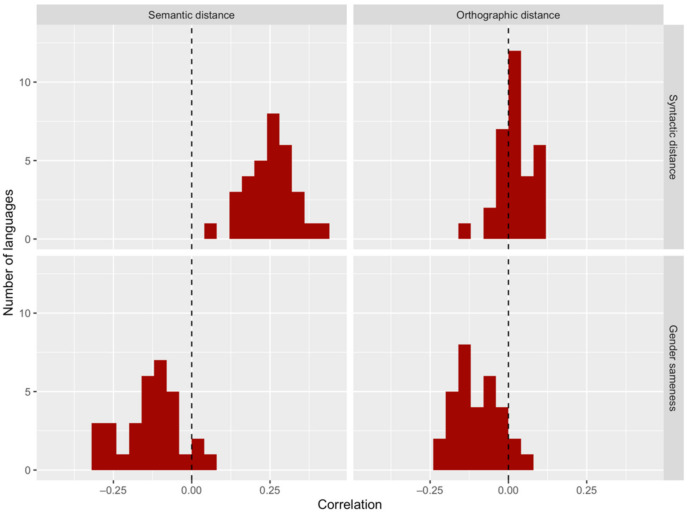
Correlations between our variables of interest (syntactic distance and gender sameness) and secondary variables (semantic distance and orthographic distance) among the 32 languages of our study. Semantic and syntactic distances are correlated positively in every language, while orthographic and syntactic distances are correlated positively in more than two-thirds of the languages. Both semantic and orthographic distances are correlated negatively with gender sameness in over 90% of the languages.

However, this approach is complicated by systematic relationships between each of these variables and secondary variables in the data: semantic and orthographic distances. The relations of syntactic distributions to both form and meaning have not been studied previously, but we offer a preview in the data presented here. The top panels of Figure 3 show the Pearson correlations between syntactic distance and both semantic and orthographic distances in the languages of our study; correlation values are shown on the *x*-axis, and the number of languages that display those correlations is shown on the *y*-axis. Syntactic and semantic distances are positively correlated in every one of these languages, while syntactic and orthographic distances are positively correlated in over two-thirds of the languages. These correlations show that, in general, syntactically similar words are also more likely to be semantically and orthographically similar.

Additionally, we know from the literature that both phonology (and its proxy orthography) and semantics are implicated in gender assignment cross-linguistically. Shared phonological patterns can indicate shared membership in a particular gender [1]. Likewise, semantically similar words have been shown to be more likely to share a gender across the lexicon [16]. These observations are borne out in our own data, as illustrated in the bottom panels of Figure 3. Both semantic and orthographic distances are correlated negatively with gender sameness in over 90 percent of our languages. These negative correlations mean that as nouns become more semantically or orthographically distant, they are less likely to share a gender.

These patterns of systematicity can help us predict the relationship that would be expected by chance between syntactic distance and gender sameness. If syntactic distance is correlated positively with both semantic and orthographic distances, and in turn these variables are both correlated negatively with gender sameness, then—all else being equal—we should also expect syntactic distance to have a negative correlation with gender sameness. Our goal is to adjust the null distribution of the correlation between syntactic distance and gender sameness to account for this systematicity elsewhere in the data. To accomplish this, we develop a variation on correlational analysis, an algorithm that we will refer to as controlled permutation.

Just like a typical permutation analysis, controlled permutation begins with a random permutation of the variable of interest—in this case the syntactic distances in the data of a particular language. However, before calculating our correlation of interest, our algorithm works incrementally to restore the known correlations between the permuted variable and the secondary variables up to a user-specified degree of tolerance (precision). Two rows of the data are chosen at random, and the algorithm evaluates whether swapping the syntactic distances of those rows would push the correlations in the desired direction. If so, the switch is made; if not, no change is made and a new pair of rows are chosen at random. These swaps continue until the original correlations with the secondary variables are restored within the desired tolerance level. In our data, the algorithm is complete when the original correlations between syntactic and both semantic and orthographic distances are restored with a tolerance of ±0.001. At that point, the correlation between syntactic distance and gender sameness is calculated. As in other permutation analyses, this process is repeated many times to create a null distribution; specifically, we conducted 10,000 controlled permutations on each language. Each simulation was performed on a sample of 10,000 rows of the data for that language, and a different sample was obtained for each simulation. We obtained one-sided *p*-values directly from the distribution, calculated as the number of correlations plus one that were greater than or equal to the true correlation, divided by the total number of correlations plus one (totaling 10,001) [55,56].

**Figure 4:**
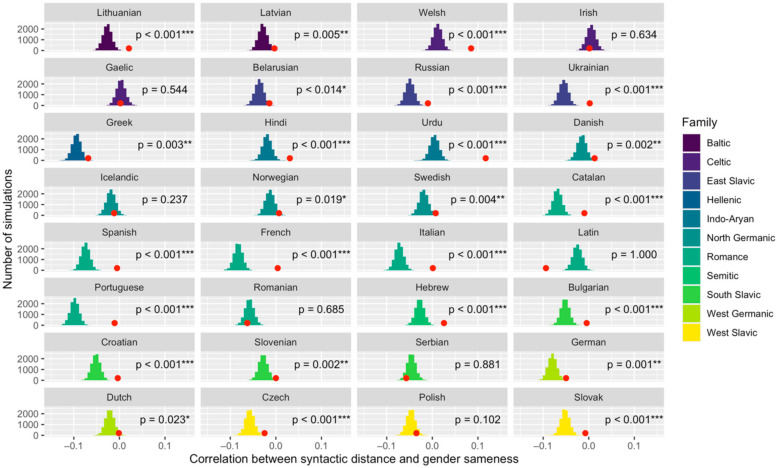
Controlled permutation analysis of the correlation between syntactic distance and gender sameness in lemma pairs of 32 languages. The red dots represent the true correlations observed in the data, while the histograms represent simulated correlations. Language families are represented by different colors. For 25 of 32 languages, the real correlation value is significantly greater than expected by chance (meaning of asterisk notation: *p* < 0.05 *; *p* < 0.01 **; *p* < 0.001 ***).

The results of the controlled permutation analysis can be found in Figure 4. We found that the correlation value between syntactic distance and probability of gender sameness is significantly greater than expected by chance in 25 out of 32 languages. Since the syntactic variable is a distance measure (rather than a measure of similarity), a greater-than-chance correlation means that syntactically similar nouns are less likely to share a gender than expected. Of the remaining seven languages, only one shows a correlation significantly lower than expected by chance. This outlier is Latin, whose status as an extinct language [57] calls into question the nature of its corpus and offers a plausible explanation for its aberrant place among these results.

It is important to note that a correlation significantly greater than chance does not necessarily mean a positive correlation. In fact, many of the significant correlations in Figure 4 are below zero. Put another way, it is not always true that syntactically similar nouns are less likely to share a gender than syntactically dissimilar nouns. For some languages the opposite is true, even if only slightly. However, when the correlation is significantly greater than chance, then we can say that syntactically similar nouns are assigned to the same gender less often than we should expect, all else being equal.

### 2.3. Mixed-Effects Regression Analysis

In addition to the permutation analysis, we fit a mixed-effects logistic regression model predicting gender sameness (*no* vs. *yes*) from orthographic, semantic, and syntactic distances and number of genders (a factor distinguishing languages with two vs. three genders). The regression was fit on randomly sampled parts of the data consisting of 10,000 pairs for each language, and each of the numeric predictors were Box–Cox normalized ([58], Section 3.4.2) and scaled within each language. We included random intercepts for each language and language family, as well as random slopes for each of the fixed effects for both of these grouping levels. This random-effects structure allows the influence of each predictor on the dependent variable to vary across languages and families. In other words, the model can reveal which effects are language- or family-specific, and which ones persist cross-linguistically. This general modeling approach follows recent studies on lexical phenomena using similarly large language samples [54,59].

Model coefficients can be found in Table 1, and they reveal the following effects for each fixed-effect predictor on the dependent variable:

- An increase in orthographic distance predicts a decrease in probability of gender sameness;
- An increase in semantic distance predicts a decrease in probability of gender sameness;
- An increase in syntactic distance predicts an increase in probability of gender sameness;
- The probability of gender sameness is lower in three-gender languages than it is in two-gender languages. (This follows logically from the principle that—all else being equal—a greater number of classes means it will be less likely that two randomly chosen elements belong to the same class.)

**Figure 5:**
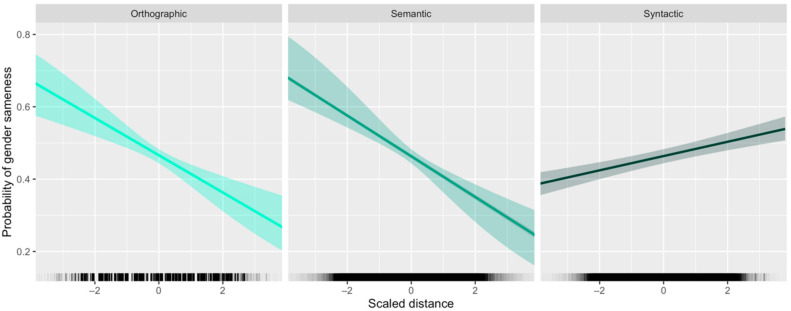
Fixed-effect plots showing the influence of orthographic, semantic, and syntactic distances on the probability of gender sameness between pairs of nouns. The first two panels show that as orthographic and semantic distances increase, probability of gender sameness decreases. The third panel shows the opposite pattern: as syntactic distance increases, probability of gender sameness also increases.

In other words, semantically and orthographically similar nouns are *more* likely to share a gender, but syntactically similar nouns are *less* likely to share a gender. These effects are illustrated in Figure 5. Inspection of the random effects indicates that the overall effect of syntactic distance is not attributable to just one or a few language families. Consistent with our correlational analysis, there is some language-specific variation in this effect, but language families do not vary substantially. Likelihood ratio tests comparing this model to four additional ones—each with one of the fixed effects removed (but no change to random effects)—indicate that the full model explains the data better than ones without a fixed effect for semantic distance (χ^2^(1) = 12.8, *p* < 0.001 ***), orthographic distance (χ^2^(1) = 11, *p* < 0.001 ***), syntactic distance (χ^2^(1) = 17.5, *p* < 0.0001 ***), and number of genders (χ^2^(1) = 19.3, *p* < 0.0001 ***).

We also want to consider the possibility that the overall effect of syntactic distance on gender sameness varies based on the number of genders in a language. Hypothetically, this effect could be strong for two-gender languages but disappear for three-gender languages, or vice versa. To test whether this is the case, we fit a model with an interaction between syntactic distance and number of genders as an additional fixed effect, along with corresponding random slopes for language and family. A likelihood ratio test comparing this new model to one without the interaction (but no change to random effects) indicates that this interaction does not significantly improve the model (χ^2^(1) = 0.025, *p* = 0.874). We can interpret this to mean that the effect of syntactic distance on gender sameness does not vary substantially between two- and three-gender languages.

## 3. Discussion

We have shown that, cross-linguistically, syntactically similar nouns are assigned to the same gender less often than syntactically distant nouns. This relationship between syntactic distance and gender sameness is exactly the opposite of the one we find for semantics and orthography. This pattern persists across a large sample of languages, and it is not driven by just one or a few languages or language families.

We interpret this finding concerning syntax as a reflection of information-theoretic pressures on language. By definition, syntactically similar words tend to occur in the same syntactic contexts, and therefore they compete against each other for activation in these contexts. A grammatical mechanism that disambiguates such words would be advantageous to language users, curbing confusability and facilitating more accurate lexical comprehension. It appears that grammatical gender serves this very role. Those syntactically similar words that compete most strongly with each other tend to be distributed across genders rather than within them. Grammatical gender has been shown to guide lexical prediction by reducing the set of candidate nouns that can occur following a gender-revealing preceding element, and we have shown that this candidate reduction process eliminates some of the strongest syntactic competitors of the target word. The apparently arbitrary system by which nouns are assigned to genders across languages may instead be a design feature of those languages.

Our findings add to a growing body of research attesting to the ways in which functional pressures on learning, memory, production, and perception shape the lexicon in different ways. The evidence for these pressures is the presence of *systematicity*: statistical patterns within a single feature—clustering or dispersion beyond chance—or correlation between two features [60]. Tendencies toward clustering and correlation can be understood within an association framework, such as the connectionist and network models of grammar in which associated items are co-activated [27]. The activation of a particular pathway benefits from having many closely related (or associated) pathways, and the compressibility that results from correlations between features is useful for learning and memory. In contrast, tendencies toward dispersion are often explained within an information-theoretic framework [17,61,62]. This perspective sees language as predictive and probabilistic, with hearers tasked with discriminating an intended message from possible alternatives. As such, it predicts maximal differentiation across lexical structures to avoid possible confusion.

There is substantial evidence supporting both of these tendencies in the lexicon, both for single features and for relationships between features. For example, clustering of phonological forms goes above and beyond the effects of morphology, homonymy, and phonotactics [63], and the resulting phonological systematicity appears to offer advantages to learning [64,65,66,67], memory [68,69], and production ([70,71,72,73,74], but cf. [75]). However, the same phonological regularity can be detrimental for perception [76,77,78], and perceptual distinctiveness is a key design feature of phonological systems [79,80,81,82]. Similarly, several studies have demonstrated a widespread correlation between form and meaning across lexicons [54,83,84,85], despite the traditional view that this relation is arbitrary [86,87]. Regular correspondences between form and meaning may facilitate learning ([83,88,89,90,91], but cf. [92,93,94]) and memory [95,96], yet they appear to cause problems for production [97,98,99]. Taken to an extreme, systematicity would lead to the presence of highly confusable words throughout the lexicon. The emerging picture is one in which patterns of systematicity in the lexicon reflect both pressures of association and dispersion.

Grammatical gender systems exhibit the same patterns we see elsewhere in the lexicon. The well documented rules relating semantic and phonological features to gender assignment reflect pressures of association. Grouping semantically and phonologically similar nouns together within genders likely serves to scaffold learning and reduce memory demands. For example, knowledge of these associations would allow a language learner to correctly infer the gender of a noun more often than in an arbitrary system. Furthermore, studies showing that speakers often know the gender of the incipient word in tip-of-the-tongue situations suggest that the association with gender may facilitate access to lexical items [100]. Yet, as we discussed earlier, the story may be more complicated with semantics. This largely taxonomic system may be interlaced with strategic exceptions in gender assignment in the form of high frequency words that aid discrimination [2].

Our primary contribution has been to further demonstrate how gender systems reflect information-theoretic pressures. Nouns are distributed among genders in such a way as to minimize confusability between targets and their syntactically similar competitors. These advantages are most salient for the hearer who is tasked with discriminating the intended message from possible alternatives. Thus, grammatical gender systems serve as a microcosm of the lexicon as whole, shaped by competing forces. Perhaps the genius of this functional negotiation is in the way opposing pressures are accommodated in different ways and in different dimensions of the lexicon. The balance of such design features suggests that language structure is evolved for efficient use [101,102]. (We leave it to future research to explore interactions among the linear predictors included in this study, and it is not clear what we should expect in such interactions. On the one hand, we might expect the discriminatory effect of syntax to be amplified when words are also semantically and/or orthographically similar, as the additional similarities add to potential confusability. On the other hand, the advantages attributed to association pressures would also be greatest under these circumstances, so it would be reasonable to predict additional clustering within genders.)

The broader research program on systematicity may also provide clues as to how these patterns in grammatical gender assignment enter and persist within the lexicon over time [60]. Diachronic explanations for such patterns are based on the understanding that words are cultural items that only persist in a language if they are efficient for communication and able to be learned [103,104,105,106]. Computational modeling and iterated language learning experiments have been employed to explore how language structures are shaped by communication between language users and transmission to new generations of language users [95,107,108,109]. This research has demonstrated how systematicity can arise through repeated cultural transmission in an initially arbitrary language [110,111]. Future research may apply similar methods to further elucidate the role of grammatical gender in disambiguating syntactically similar words.

## Figures and Tables

**Table 1:** Coefficients of the mixed-effects generalized linear regression model predicting gender sameness for pairs of nouns in 32 languages.

	β	95% CI (Lower)	95% CI (Upper)	SD (Family)	SD (Language)
Intercept	0.202	0.060	0.344	0.000	0.002
Orthographic distance	−0.217	−0.315	−0.119	0.159	0.067
Semantic distance	−0.277	−0.387	−0.166	0.169	0.121
Syntactic distance	0.081	0.053	0.109	0.000	0.078
Number of genders (2)	-	-	-	0.153	0.148
Number of genders (3)	−0.745	−0.897	−0.592	0.012	0.096

## Data Availability

All code for this study are available in a public GitHub repository at https://github.com/pgr179/grammatical_gender_disambiguates_syntactically_similar_words (accessed on 31 March 2022).
